# A novel method to test associations between a weighted combination of phenotypes and genetic variants

**DOI:** 10.1371/journal.pone.0190788

**Published:** 2018-01-12

**Authors:** Huanhuan Zhu, Shuanglin Zhang, Qiuying Sha

**Affiliations:** Department of Mathematical Sciences, Michigan Technological University, Houghton, Michigan, United States of America; Indiana University Bloomington, UNITED STATES

## Abstract

Many complex diseases like diabetes, hypertension, metabolic syndrome, et cetera, are measured by multiple correlated phenotypes. However, most genome-wide association studies (GWAS) focus on one phenotype of interest or study multiple phenotypes separately for identifying genetic variants associated with complex diseases. Analyzing one phenotype or the related phenotypes separately may lose power due to ignoring the information obtained by combining phenotypes, such as the correlation between phenotypes. In order to increase statistical power to detect genetic variants associated with complex diseases, we develop a novel method to test a weighted combination of multiple phenotypes (WCmulP). We perform extensive simulation studies as well as real data (COPDGene) analysis to evaluate the performance of the proposed method. Our simulation results show that WCmulP has correct type I error rates and is either the most powerful test or comparable to the most powerful test among the methods we compared. WCmulP also has an outstanding performance for identifying single-nucleotide polymorphisms (SNPs) associated with COPD-related phenotypes.

## Introduction

Genome-wide association studies (GWAS) aim to discover genetic variants associated with complex diseases [1, 2]. In GWAS, researchers often collect data on multiple correlated phenotypes to get a better understanding of the complex disease [3]. Here are some examples of what diseases are measured by multiple phenotypes. In type 2 diabetes (T2D) studies data are usually collected on a number of risk factors and diabetes-related quantitative phenotypes. Hypertension is measured by systolic blood pressures (SBP) and diastolic blood pressures (DBP) [2], and the correlation coefficient between SBP and DBP was greater than 0.5 in 95% of patients [4]. The metabolic syndrome refers to the co-occurrence of insulin resistance, obesity, atherogenic dyslipidemia and hypertension, and these factors are associated and share underlying mediators, pathway and mechanisms [5]. The correlations between multiple phenotypes can be leveraged to improve the power of genetic association tests to identify markers associated with one or more of the phenotypes [6]. The standard approach to analyze these multiple correlated phenotypes is to perform single-phenotype analyses separately and report the findings for each phenotype [1]. However, analyzing one phenotype at a time will suffer penalties from the multiple testing and result in a reduced power especially for GWAS [3]. Recently, the joint analysis of multiple phenotypes has become popular because it can increase statistical power over analyzing phenotypes separately in detecting genetic variants [3, 6].

There are three commonly used strategies to detect genetic associations between a genetic variant and multiple correlated phenotypes. The first one is combining test statistics (or p-values) from univariate analysis. This strategy first tests an association between each phenotype and a genetic variant individually and then combines the univariate analysis results, i.e. test statistics or p-values, by using different approaches. The O’Brien’s method [7], sample splitting and cross-validation method [3], Trait-based Association Test that uses Extended Simes procedure (TATES) [8], Unified Score-Based Association Test (USAT) [9], Fisher’s Combination [10], and Adaptive Fisher’s Combination (AFC) [11] belong to this strategy. The advantage of this strategy is its simplicity and is especially useful for analyzing different types of phenotypes such as continuous, dichotomous and survival [2]. The second one is data reduction. This strategy derives a single or a few new phenotypes that are linear combinations of the original phenotypes. Existing methods include projection-based techniques and canonical correlation analysis (CCA). Projection-based approaches include principal components analysis (PCA) and principal component of heritability (PCH), where principal components (PCs) are built to maximize either the phenotypic variance or heritability [2, 6, 12, 13]. Canonical correlation analysis (CCA) finds the linear combination of phenotypes that explain the largest possible amount of the correlation between the genetic variant and all multiple phenotypes [14]. Data reduction approaches are in general only applicable to multiple phenotypes consisting of all continuous phenotypes that are approximately normally distributed [2]. The third strategy is regression models which include mixed effect models [15–17], the generalized estimating equation (GEE) [18, 19], and reverse regression methods [1, 20, 21]. The linear mixed effects model (LME) and generalized linear mixed effects model (GLMM) are two commonly used mixed effects models, where the fixed effects are used for the genetic variant and random effects are used to account for phenotypic correlations. The GEE methods collapse the random effects and random residual errors in marginal regression models which are a class of models different from mixed effect models. The reverse regression methods take genotypes as the response variable and multiple phenotypes as predictors, such as the proportional odds logistic regression for joint model of multiple phenotypes (MultiPhen) [1]. Regression approaches are able to deal with a mixture of continuous, dichotomous, and survival phenotypes, but they are complicated and few available software were developed to implement these methods [2].

In this article, we developed a novel allele-based method for testing association between multiple phenotypes and a genetic variant. First, we take the allele at the genetic variant as the response variable and the multiple phenotypes as predictors. Then, we present a new multivariate method that we refer to as WCmulP (Weighted Combination of multiple Phenotypes), inspired by TOW (Test for testing the effect of an Optimally Weighted combination of variants) procedure proposed by Sha et al. [22] for rare variant association studies and allele-based aproach proposed by Majumdar et al. [23]. For each of the independent individuals, WCmulP linearly combines the multiple phenotypes to “one phenotype” by using the optimal weights proposed by Sha et al. [22]. Then we use the score test based on the logistic model to test the association between the genetic variant and the linear combination of phenotypes. Using extensive simulation studies, we compare the performance of WCmulP with some of the existing methods, MultiPhen[1], O’Brien’s method [7], TATES [8], CCA [14], and SHet [24]. Our results show that, in all of the simulation scenarios, WCmulP is either the most powerful test or comparable to the most powerful tests among the methods we compared. Finally, we evaluate the performance of our proposed method using a real data set, the COPDGene study from dbGaP.

## Methods

We consider a sample of *n* unrelated individuals. Each individual has *K* possibly correlated phenotypes. Let *Y*_*i*,*k*_ denote the *k*^*th*^ phenotype of the *i*^*th*^ individual. We propose to use an allele-based logistic regression model to test the association between a variant of interest and multiple phenotypes. For a genetic variant with two alleles, we use *x*_2*i*−1_ and *x*_2*i*_ to denote the coding of the two alleles of the *i*^th^ individual such that we use *x*_1_ and *x*_2_ to code the two alleles of the first individual, use *x*_3_ and *x*_4_ to code the two alleles of the second individual, and so on. For a variant with two alleles *A* and *a*, if the genotype of the *i*^*th*^ individual is *AA*, we define *x*_2*i*−1_ = *x*_2*i*_ = 1; if the genotype is *aa*, we define *x*_2*i*−1_ = *x*_2*i*_ = 0; and if the genotype is *Aa*, we define *x*_2*i*−1_ = 1; and *x*_2*i*_ = 0. We define the *k*^*th*^ phenotype corresponding to the two alleles *x*_2*i*−1_ and *x*_2*i*_ of the *i*^*th*^ individual as *y*_2*i*−1,*k*_ and *y*_2*i*,*k*_, where *y*_2*i*−1,*k*_ = *y*_2*i*,*k*_ = *Y*_*i*,*k*_. Hence, the total number of observations in the allele-based data is 2*n*. We model the relationship between alleles and multiple phenotypes using the inverse logistic regression model

$$\mathrm{logit}\left(\pi_{j}\right)=\alpha +y_{j,1}\beta_{1}+y_{j,2}\beta_{2}+\cdots +y_{j,K}\beta_{K},\;j=1,2,\ldots ,2n,$$ (1)

where *π*_*j*_ = Pr(*x*_*j*_ = 1|***Y***_*j*_ = (*y*_*j*,1_,…,*y*_*j*,*K*_)^*T*^), *α* is the intercept, and ***β*** = (*β*_1_,…,*β*_*K*_)^*T*^ is a *K*-dimention vector of parameters. To test the association between multiple phenotypes and the variant is equivalent to test the null hypothesis *H*_0_: ***β* = 0** under Eq (1). We use the score test statistic given by Sha et al. [25] to test *H*_0_: ***β* = 0** under Eq (1). The test statistic is

$$S=U^{T}V^{-1}U,$$ (2)

where $U=\sum_{j=1}^{2n}(x_{j}-\bar{x})Y_{j},V=(1-\bar{x})\bar{x}\;\sum_{j=1}^{2n}(Y_{j}-\bar{Y}){\left(Y_{j}-\bar{Y}\right)}^{T},\bar{x}=\frac{1}{2n}\sum_{j=1}^{2n}x_{j},\;\bar{Y}={\left({\bar{y}}_{1},\ldots ,{\bar{y}}_{K}\right)}^{T}$ and ${\bar{y}}_{k}=\frac{1}{2n}\sum_{j=1}^{2n}y_{j,k}$ for *k* = 1,…,*K*. The test statistic *S* asymptotically follows a chi-square distribution with *K* degrees of freedom.

When *K* is large, the score test may lose power due to the large degrees of freedom. To overcome this problem, we combine the *K* phenotypes to one variable by using a linear combination of phenotypes, $y_{j}=\sum_{k=1}^{K}w_{k}y_{j,k}$, where *w*_1_,…, *w*_*K*_ are the weights. With the linear combination of phenotypes $y_{j}=\sum_{k=1}^{K}w_{k}y_{j,k}$, the score test statistic in Eq (2) becomes

$$S\left(w_{1},\ldots ,w_{K}\right)=2n\frac{{\left(\sum_{j=1}^{2n}\left(x_{j}-\bar{x}\right)y_{j}\right)}^{2}}{\sum_{j=1}^{2n}{\left(x_{j}-\bar{x}\right)}^{2}\sum_{j=1}^{2n}{\left(y_{j}-\bar{y}\right)}^{2}}.$$ (3)

We propose to use the optimal weights proposed by Sha et al. [22], that is, $w_{k}^{o}=\frac{\sum_{j=1}^{2n}\left(x_{j}-\bar{x})(y_{j,k}-{\bar{y}}_{k}\right)}{\sum_{j=1}^{2n}{\left(y_{j,k}-{\bar{y}}_{k}\right)}^{2}}$ for *k* = 1,2, …, *K*. Actually, the optimal weights $w_{1}^{o},\ldots ,w_{K}^{o}$ maximize *S*(*w*_1_,…, *w*_*K*_) in Eq (3). With this optimally weighted combination of phenotypes $y_{j}^{o}=\sum_{k=1}^{K}w_{k}^{o}y_{j,k}$, the test statistic given in Eq (3) becomes

$$S\left(w_{1}^{o},\ldots ,w_{K}^{o}\right)=2n∙\frac{\sum_{j=1}^{2n}\left(x_{j}-\bar{x}\right)\left(y_{j}^{o}-{\bar{y}}^{o}\right)}{\sum_{j=1}^{2n}{\left(x_{j}-\bar{x}\right)}^{2}},$$ (4)

where ${\bar{y}}^{o}=\frac{1}{2n}\sum_{j=1}^{2n}y_{j}^{o}$. From Eq (2)–Eq (4), we reduced the dimension of the phenotypes from multivariate (*y*_*j*,*k*_, *k* = 1, …, *K*) to univariate $\left(y_{j}^{o}\right)$ with optimal weights $w_{k}^{o}$ such that Eq (4) is the maximum of Eq (3). Since $w_{1}^{o},\ldots ,w_{K}^{o}$ are data-driven weights, $S(w_{1}^{o},\ldots ,w_{K}^{o})$ does not follow a chi-square distribution. We use a permutation procedure to evaluate the p-value of $S(w_{1}^{o},\ldots ,w_{K}^{o})$. In each permutation, we randomly shuffle the genotypes and keep the phenotypes unchanged. Since $\sum_{j=1}^{2n}{\left(x_{j}-\bar{x}\right)}^{2}$ does not change under each permutation, the test statistic $S(w_{1}^{o},\ldots ,w_{K}^{o})$ is equivalent to

$$T=\sum_{j=1}^{2n}\left(x_{j}-\bar{x}\right)\left(y_{j}^{o}-{\bar{y}}^{o}\right).$$ (5)

This test statistic *T* is our proposed test statistic to test the effect of the Weighted Combination of multiple Phenotypes (WCmulP).

The WCmulP method can also be extended to incorporate covariates. Suppose that there are *p* covariates. Let *Z*_*i*,*l*_ denote the *l*^*th*^ covariate of the *i*^*th*^ individual. We define the *l*^*th*^ covariate corresponding to the two alleles *x*_2*i*−1_ and *x*_2*i*_ of the *i*^*th*^ individual as *z*_2*i*−1,*l*_ and *z*_2*i*,*l*_, where *z*_2*i*−1,*l*_ = *z*_2*i*,*l*_ = *Z*_*i*,*l*_. We then adjust the phenotype value *y*_*j*,*k*_ for the covariates by applying linear regressions. That is,

$$y_{j,k}=\alpha_{0,k}+\alpha_{1,k}z_{j,1}+\cdots +\alpha_{p,k}z_{j,p}+\tau_{j,k}.$$

Let ${\tilde{y}}_{j,k}$ denote the residuals of *y*_*j*,*k*_ in the linear regression. We incorporate the covariate effects in WCmulP by replacing *y*_*j*,*k*_ in Eq (5) by ${\tilde{y}}_{j,k}$. With covariates, the statistic of WCmulP is defined as

$$T_{WCmulP}={T|}_{y_{j,k}={\tilde{y}}_{j,k}}.$$

## Comparison of methods

We compare the power of the proposed WCmulP with that of the following methods:

**Score** (Score test): the test statistic of Score is given by Eq (2).

**OB** (O’Brien’s method) [7]: the test statistic of OB, ***e***^*T*^**Σ**^−1^***T***_uni_, is a linear combination of univariate test statistics, and it is the most powerful test among a class of test statistics that are linear combination of ***T***_uni_, where ***T***_uni_ is the vector of the univariate test statistics, Σ is the covariance matrix of ***T***_uni_, and ***e*** = (1,1…,1)^*T*^ is a 1’s vector with length *K* (the number of phenotypes).

**MultiPhen** (Joint model of Multiple Phenotypes) [1]: it uses the proportional odds logistic regression to model the genotype data as ordinal response and phenotypes as predictors. A likelihood ratio test is used to test the null hypothesis.

**TATES** (Trait-based Association Test that uses Extended Simes procedure) [8]: it combines univariate p-values to acquire one phenotype-based p-value, while correcting for correlations between phenotypes. The TATES p-value is given by $Min\;\left(\frac{m_{e}p_{\left(k\right)}}{m_{e(k)}}\right)$, where *p*_(*k*)_ is the *k*^*th*^ (*k* = 1,…,*K*) sorted p-value in ascending order, *m*_*e*_ and *m*_*e*(*k*)_ are the effective numbers of independent p-values of all *K* phenotypes and *k* specified phenotypes, respectively. The effective numbers can be calculated from the correlation matrix of p-values.

**CCA** (Canonical Correlation Analysis) [14]: it extracts the linear combination of phenotypes that maximizes the correlations between linear combinations of phenotypes and genotypes at the variant of interest. The test is based on Wilks’ lambda and the corresponding F-approximation.

**SHet** (Test for Heterogeneous genetic effects) [24]: The test statistic of SHet, *S*_*Het*_, is based on *S*_*Hom*_, which is the most powerful test statistic when the genetic effect is homogeneous. Both *S*_*Hom*_ and *S*_*Het*_ are quadratic combinations of the univariate test statistics. The test statistic of *S*_*Hom*_ is $S_{Hom}=\frac{e^{T}{\left(RW\right)}^{-1}\;{T_{\mathrm{uni}}(e^{T}{\left(RW\right)}^{-1}T_{\mathrm{uni}})}^{T}}{e^{T}{\left(WRW\right)}^{-1}e}$, where *R* is the correlation matrix of ***T***_uni_, *W* is a diagonal matrix of weights for the univariate test statistics, and *e* is a 1’s vector with length *K* (number of phenotypes). *S*_*Het*_ can be viewed as the maximum of *S*_*Hom*_’s satisfying different thresholds. More specifically, given a threshold, only test statistics with absolute values that are greater than the threshold are used, *R* and *W* are therefore partially used corresponding to the selected test statistics. The p-values of *S*_*Het*_ can be evaluated by simulation.

## Simulation studies

Our simulations are similar to that of Wang et al. [13]. To evaluate the type I error rates and powers of our method, we simulate genotype-phenotype data sets for *n* unrelated individuals with total *K* phenotypes according to a variety of simulation scenarios. Specifically, genotype data at a genetic variant are simulated according to the minor allele frequency (MAF) under the assumption of Hardy-Weinberg equilibrium. We generate *K* phenotypes by the factor model

$$y=\lambda x+c\gamma f+\sqrt{1-c^{2}}\times \varepsilon ,$$ (6)

where *y* = (*y*_1_,…,*y*_*K*_)^*T*^; *x* is the genotype score at the variant of interest; *λ* = (*λ*_1_,…,*λ*_*K*_) is the vector of effect sizes of the genetic variant on the *K* phenotypes; *f* = (*f*_1_,…,*f*_*R*_)^*T*^ ∼ *MVN*(0,Σ), Σ = (1−*ρ*)*I* + *ρA*, *R* is the number of factors, *A* is a matrix with elements of 1, *I* is the identity matrix, and *ρ* is the correlation between *f*_*i*_ and *f*_*j*_ for *i* ≠ *j*; **γ** is a *K* by *R* matrix; *c* is a constant number; and *ε* = (*ε*_1_,…,*ε*_*K*_)^*T*^ is a vector of residuals, *ε*_1_,…,*ε*_*K*_ are independent, and *ε*_*k*_ ∼ *N*(0,1) for *k* = 1,…,*K*. Based on Eq (6), we consider the following six models.

**Model 1**: There is only one factor and genotype has an impact on all traits with the same effect size. That is, *R* = 1, *λ* = (*β*,…,*β*)^*T*^, and **γ** = (1,…,1)^*T*^.

**Model 2**: There are two factors and genotype has an impact on two factors with opposite effects. That is, *R* = 2, $\lambda ={\left(\underset{K/2}{\underset{︸}{-\beta ,\ldots ,-\beta }},\underset{K/2}{\underset{︸}{\beta ,\ldots ,\beta }}\right)}^{T}$, and *γ* = *bdiag*(*D*_1_,*D*_2_), where $D_{i}={\left(\underset{K/2}{\underset{︸}{1,\ldots ,1}}\right)}^{T}$ for *i* = 1,2, “*bdiag*” indicates the block diagonal matrix.

**Model 3**: There are two factors and genotype has an impact on one factor. That is, *R* = 2, $\lambda ={\left(0,\ldots ,0,\underset{K/2}{\underset{︸}{\beta ,\ldots ,\beta }}\right)}^{T}$, and *γ* = *bdiag*(*D*_1_,*D*_2_), where $D_{i}={\left(\underset{K/2}{\underset{︸}{1,\ldots ,1}}\right)}^{T}$ for *i* = 1,2.

**Model 4**: There are four factors and genotype has an impact on one factor. That is, *R* = 4, $\lambda ={\left(0,\ldots ,0,\underset{K/4}{\underset{︸}{\beta ,\ldots ,\beta }}\right)}^{T}$, and *γ* = *bdiag*(*D*_1_,*D*_2_,*D*_3_,*D*_4_), where $D_{i}={\left(\underset{K/4}{\underset{︸}{1,\ldots ,1}}\right)}^{T}$ for *i* = 1,…,4.

**Model 5**: There are four factors and genotype has an impact on two factors with opposite effects. That is, *R* = 4, $\lambda ={\left(0,\ldots ,0,\underset{K/4}{\underset{︸}{-\beta ,\ldots ,-\beta }},\underset{K/4}{\underset{︸}{\beta ,\ldots ,\beta }}\right)}^{T}$, and *γ* = *bdiag*(*D*_1_,*D*_2_,*D*_3_,*D*_4_), where $D_{i}={\left(\underset{K/4}{\underset{︸}{1,\ldots ,1}}\right)}^{T}$ for *i* = 1,…,4.

**Model 6**: There are four factors and genotype has an impact on three factors with effects of different directions. That is, *R* = 4, $\lambda ={\left(0,\ldots ,0,\frac{2\beta }{K/4+1}\times 1,\frac{2\beta }{K/4+1}\times 2,\ldots ,\frac{2\beta }{K/4+1}\times \frac{K}{4},\underset{K/4}{\underset{︸}{-\beta ,\ldots ,-\beta }},\underset{K/4}{\underset{︸}{\beta ,\ldots ,\beta }}\right)}^{T}$, and *γ* = *bdiag*(*D*_1_,*D*_2_,*D*_3_,*D*_4_), where $D_{i}={\left(\underset{K/4}{\underset{︸}{1,\ldots ,1}}\right)}^{T}$ for *i* = 1,…,4.

In the six models, the within-factor correlation is *c*^2^ and the between-factor correlation is *ρc*^2^. Table A in S1 File gives the structures of *γ* and cov(*y*|*x*) for different numbers of factors (*R* = 1,2, and 4) when the number of phenotypes is 8.

We also generate phenotypes with covariates effects. We refer to Sha et al. [22] and Sun et al. [26] by adding two covariates in Eq (6) as $y=\left(0.5z_{1}+0.5z_{2}\right)e+\lambda x+c\gamma f+\sqrt{1-c^{2}}\times \varepsilon$, where *z*_1_ is a continuous random variable generated from a standard normal distribution, *z*_2_ is a binary random variable taking values of 0 and 1 with a probability of 0.5, and *e* is a K-dimensional vector with all elements being 1’s. To evaluate type I error rates and powers, we consider *n* = 1,000 unrelated individuals, *MAF* = 0.3, and different numbers of phenotypes *K* = 8,16. To evaluate the type I error rates of all methods, we generate all phenotypes independent of genotypes by setting *β* = 0. We evaluate type I error rates at significance levels *α* = 0.001 and 0.01 for all methods. To evaluate powers, we vary the values of *β* (within-factor correlation *c*^2^ = 0.5 and between-factor correlation *ρc*^2^ = 0.1) and vary the values of within-factor correlation *c*^2^ (0.3,0.5,…,0.9) (between-factor correlation *ρc*^2^ = 0.1 and *β* = 0.1,).

## Simulation results

To evaluate the type I error rates of WCmulP and other six methods, we consider different numbers of phenotypes, different significance levels, and different numbers of factors. In each simulation scenario, the p-values of WCmulP and SHet are estimated using 10,000 permutations, and the p-values of Score, MultiPhen, TATES, CCA and OB are estimated using their asymptotic distributions. The type I error rates of the seven methods are evaluated using 10,000 replicated samples. For 10,000 replicated samples, the 95% confidence intervals (CIs) for type I error rates of nominal levels 0.001 and 0.01 are (0.00038,0.00162) and (0.008,0.012), respectively. The estimated type I error rates of WCmulP and other six methods are summarized in Table 1 (*K* = 8) and Table 2 (*K* = 16). From these tables, we can see that all estimated type I error rates of WCmulP are within 95% CIs, which indicates that the proposed WCmulP is a valid test. The estimated type I error rates of SHet, Score, MultiPhen, TATES, CCA and OB are not significantly different from the nominal levels.

**Table 1:** Estimated type I error rates for the seven methods under three simulation settings. The number of phenotypes is *K* = 8, *c*^2^ = 0.5, *ρc*^2^ = 0.1, and *MAF* = 0.3. The p-values of WCmulp and SHet are evaluated using 10,000 permutations. The type I error rate of all of the seven methods is evaluated using 10,000 replicated samples at a significance level of *α*. *R* is the number of factors.

			Type I error rates			
	*R* = 1		*R* = 2		*R* = 4	
Methods	*α* = 0.001	*α* = 0.01	*α* = 0.001	*α* = 0.01	*α* = 0.001	*α* = 0.01
WCmulP	0.0008	0.0097	0.0011	0.0091	0.0011	0.0104
SHet	0.0008	0.0106	0.0009	0.0093	0.0008	0.0104
Score	0.0006	0.0102	0.0008	0.0103	0.0004	0.0105
MultiPhen	0.0011	0.0106	0.0011	0.0105	0.0005	0.0107
TATES	0.0012	0.0094	0.0007	0.0121	0.0004	0.0106
CCA	0.0008	0.0107	0.0010	0.0099	0.0008	0.0107
OB	0.0007	0.0095	0.0016	0.0092	0.0013	0.0105

**Table 2:** Estimated type I error rates for the seven methods under three simulation settings. The number of phenotypes is *K* = 16, *c*^2^ = 0.5, *ρc*^2^ = 0.1, and *MAF* = 0.3. The p-values of WCmulp and SHet are evaluated using 10,000 permutations. The type I error rate of all of the seven methods is evaluated using 10,000 replicated samples at a significance level of *α*.

			Type I error rates			
	*R* = 1		*R* = 2		*R* = 4	
Methods	*α* = 0.001	*α* = 0.01	*α* = 0.001	*α* = 0.01	*α* = 0.001	*α* = 0.01
WCmulP	0.0011	0.0089	0.0006	0.0094	0.0008	0.0098
SHet	0.0009	0.0098	0.0009	0.0126	0.0008	0.0088
Score	0.0010	0.0096	0.0011	0.0098	0.0010	0.0086
MultiPhen	0.0011	0.0096	0.0011	0.0121	0.0013	0.0103
TATES	0.0013	0.0110	0.0012	0.0102	0.0008	0.0104
CCA	0.0012	0.0097	0.0009	0.0111	0.0011	0.0089
OB	0.0011	0.0085	0.0006	0.0092	0.0007	0.0097

**Fig 1:**
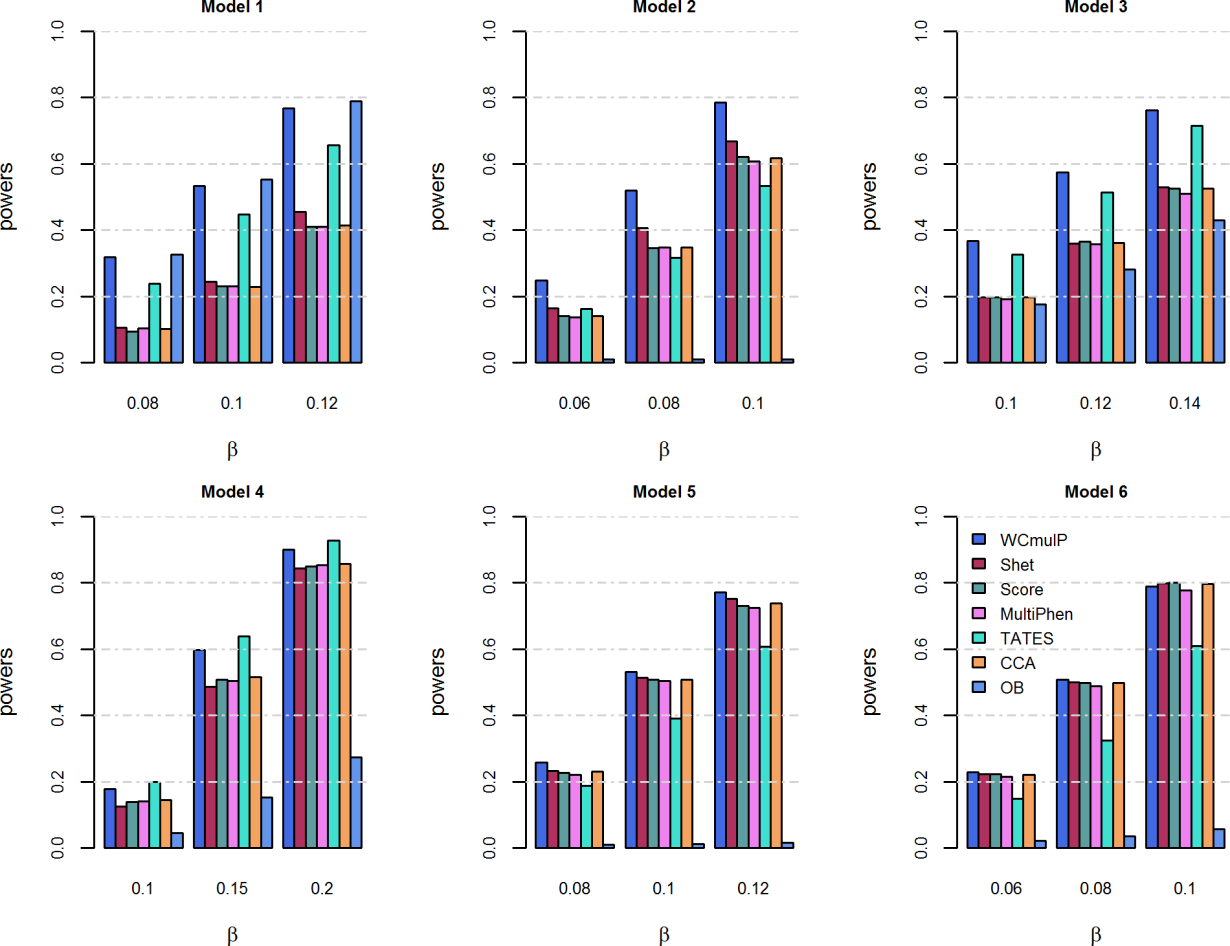
Power comparisons of the seven methods as a function of *β* for the six models. The total number of phenotypes is *K* = 8, *c*^2^ = 0.5, *ρc*^2^ = 0.1, and *MAF* = 0.3. The p-values of WCmulP and SHet are evaluated using 1,000 permutations. The power of all of the seven methods is evaluated using 1,000 replicated samples at a significance level of 0.01.

**Fig 2:**
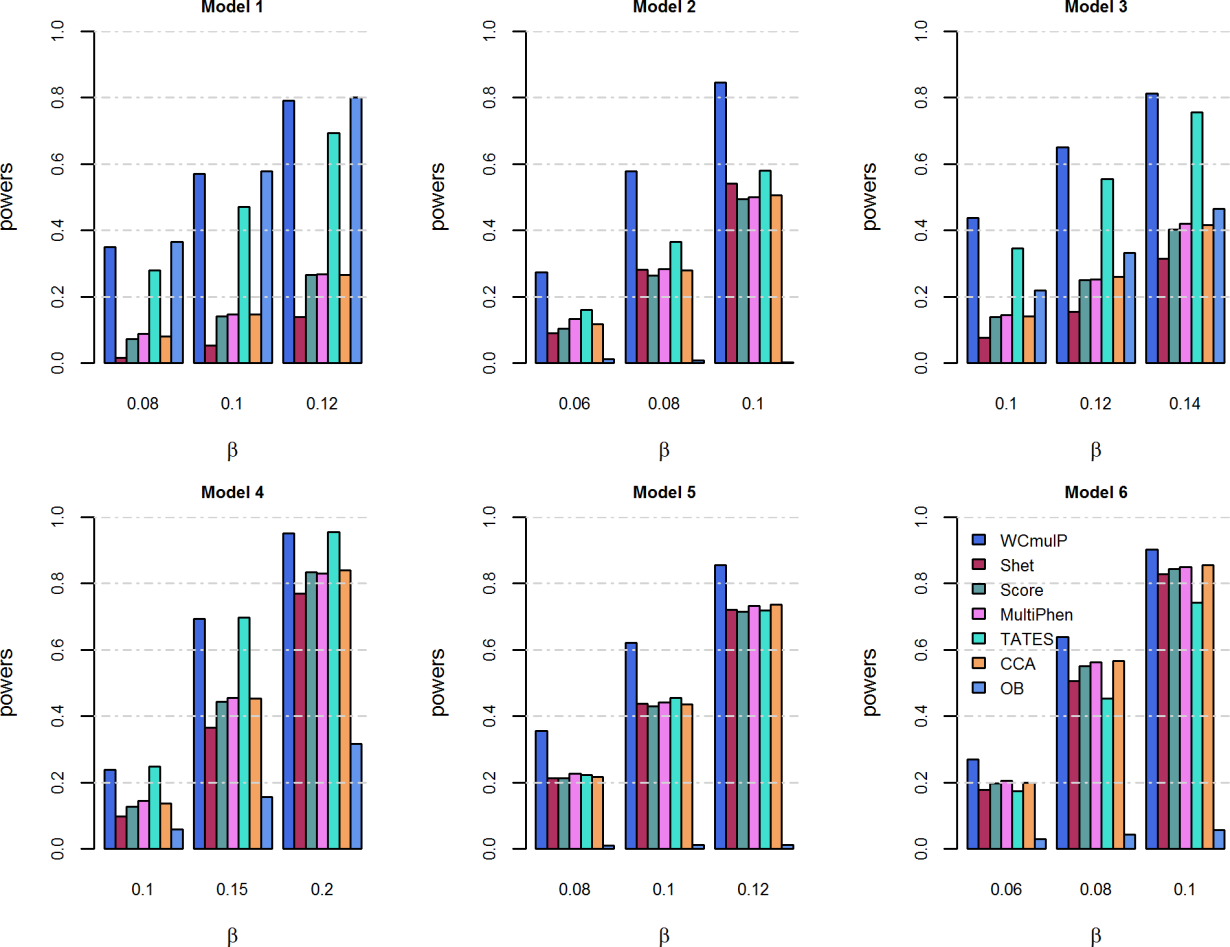
Power comparisons of the seven methods as a function of *β* for the six models. The total number of phenotypes is *K* = 16, *c*^2^ = 0.5, *ρc*^2^ = 0.1, and *MAF* = 0.3. The p-values of WCmulP and SHet are evaluated using 1,000 permutations. The power of all of the seven methods is evaluated using 1,000 replicated samples at a significance level of 0.01.

**Fig 3:**
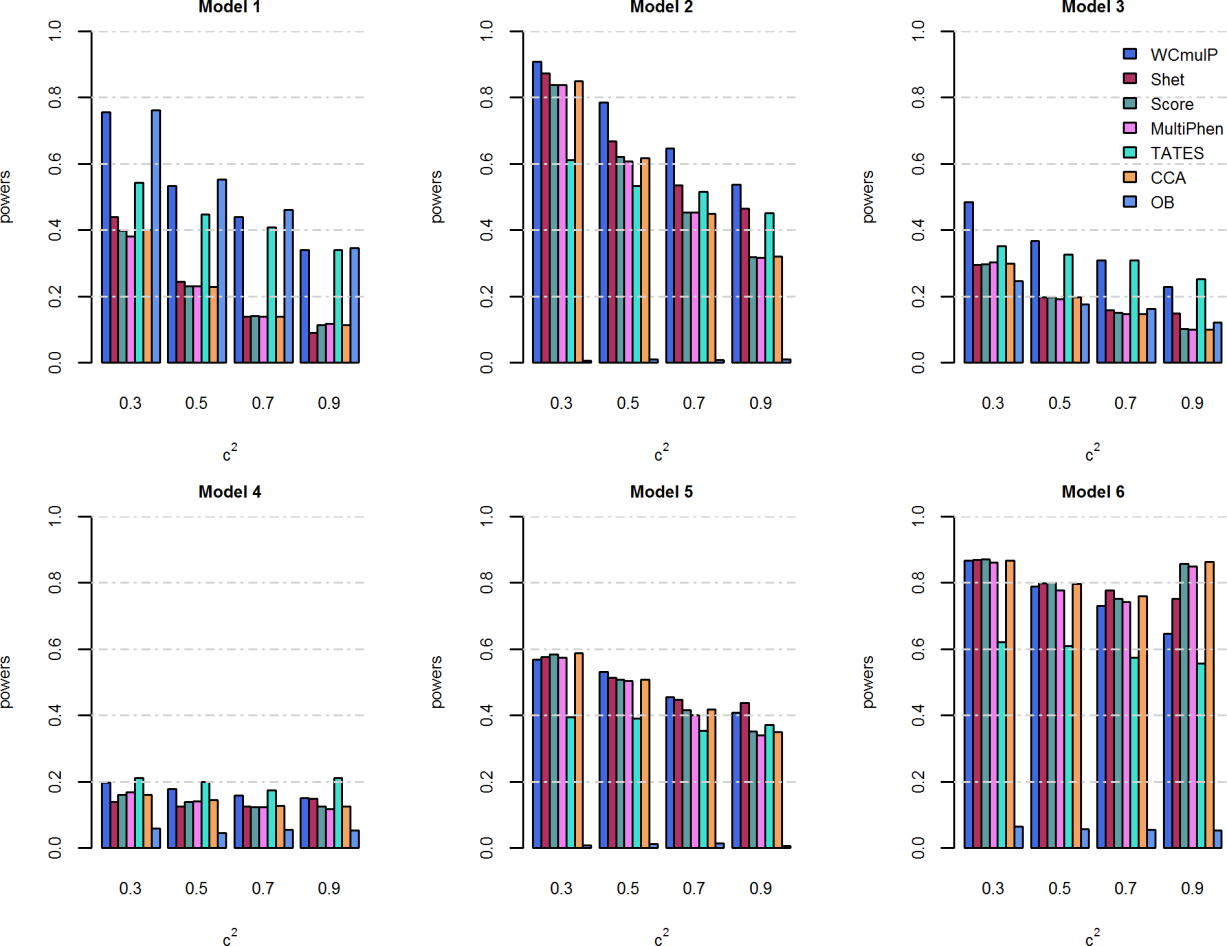
Power comparisons of the seven methods as a function of *c*^2^ for the six models. The total number of phenotypes is *K* = 8, *ρc*^2^ = 0.1, *β* = 0.1, and *MAF* = 0.3. The p-values of WCmulP and SHet are evaluated using 1,000 permutations, the p-values of other methods are evaluated using asymptotic distribution. The power of all of the seven methods is evaluated using 1,000 replicated samples at a significance level of 0.01.

**Fig 4:**
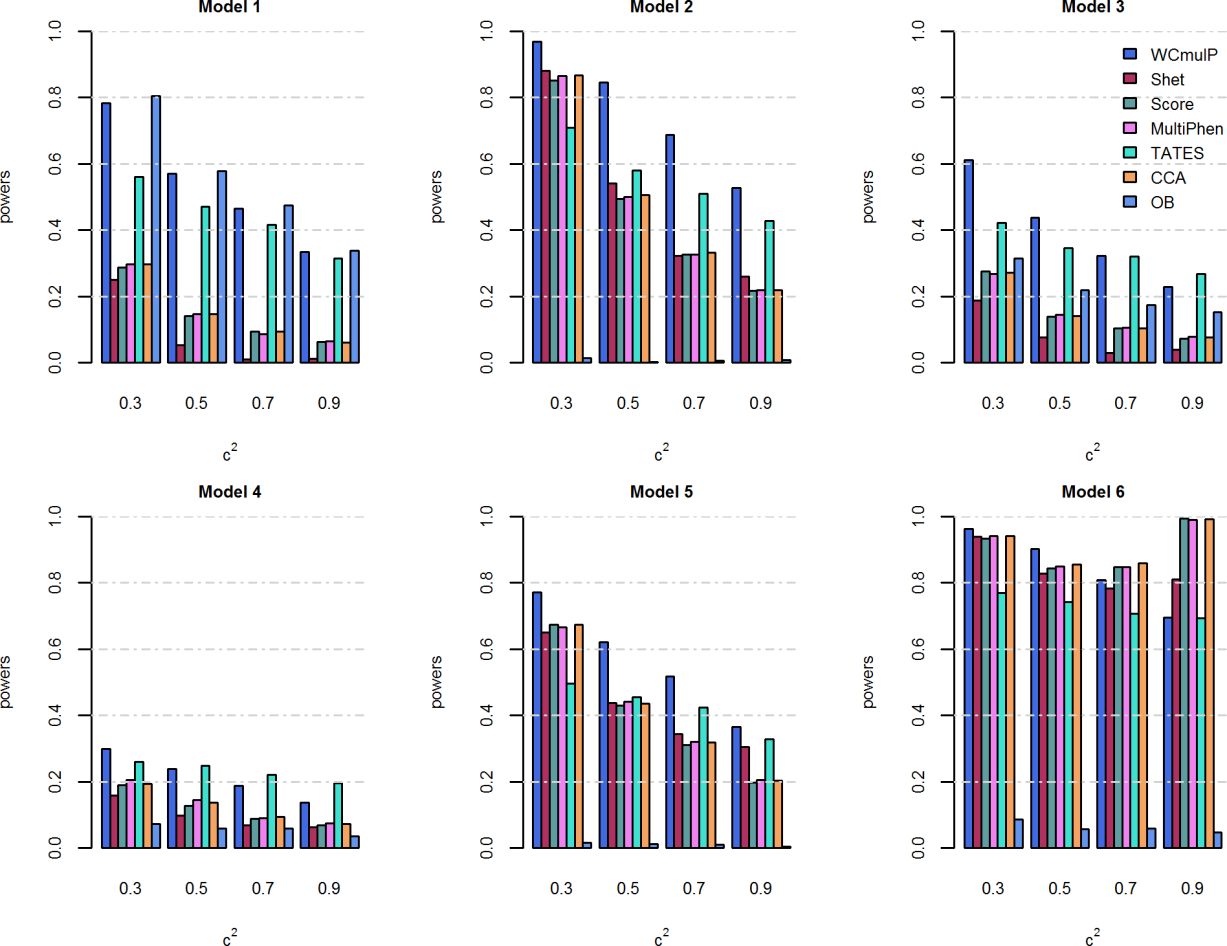
Power comparisons of the seven methods as a function of *c*^2^ for the six models. The total number of phenotypes is *K* = 16, *ρc*^2^ = 0.1, *β* = 0.1, and *MAF* = 0.3. The p-values of WCmulP and SHet are evaluated using 1,000 permutations, the p-values of other methods are evaluated using asymptotic distribution. The power of all of the seven methods is evaluated using 1,000 replicated samples at a significance level of 0.01.

For power comparisons, we consider power as a function of genetic effect *β* (Figs 1 and 2) and power as a function of within-factor correlation *c*^2^ (Figs 3 and 4). In each of the simulation scenario, the p-values of WCmulP and SHet are estimated using 1,000 permutations and the p-values of Score, MultiPhen, TATES, CCA and OB are estimated using their asymptotic distributions. The powers of the seven methods are evaluated using 1,000 replicated samples at a significance level of 0.01.

Our simulation results show that:

1. As expected, the powers of all methods increase as the genetic effect *β* increases in each model (Figs 1 and 2).
2. WCmulP is either the most powerful test or comparable to the most powerful tests in all six models (Figs 1–4).
3. As number of phenotypes increases from *K* = 8 to *K* = 16, WCmulP presents more obvious ascendancy than other methods.
4. SHet, Score, MultiPhen, and CCA have similar performance in all six models; we call these four tests as group 1.
5. OB is the most powerful test when the genetic effects are homogeneous (model 1). However, OB reduces power significantly when genetic effects are heterogeneous, especially when opposite directions of the genetic effects exist (models 2, 5–6) or when the genetic variant impacts only a small portion of phenotypes (model 4). This phenomenon was also observed by Zhu et al. [27].
6. Power comparisons of TATES with tests in group 1 depend on the models. In general, TATES is more powerful than tests in group 1 when the genetic variant impacts on a portion of phenotypes (models 3 and 4).
7. In general, as the within-factor correlation *c*^2^ increases, the powers of all methods decrease (Figs 3 and 4). TATES is relatively robust to *c*^2^ because it essentially only depends on the phenotype that has the strongest association with the genetic variant, as explained in Zhu et al. [27].

We also considered using principal components (PCs) of the phenotypes instead of the original phenotypes to do power comparisons and the results are given in Figures A-D in S1 File. We exclude PCs that explain less than 10^−6^ of the total variation. Using PCs of the phenotypes, we observe that: (1) WCmulP, Score, MultiPhen, and CCA have very similar powers in all six models (Figures A-D in S1 File). We call these tests as group s1. The tests in group s1 are either the most powerful tests or comparable to the most powerful one; (2) SHet is less powerful than the tests in group s1; (3) OB is the least powerful method in all six models because PCs likely have effects with different directions; (4) TATES becomes the most powerful method when the genetic variant has effects on all phenotypes with the same absolute value of effect sizes (models 1 and 2) because in this case, one of the PCs may capture the most of association information.

We also compared the powers using a lower significance level 5×10^−5^ (Figure E in S1 File). Figure E in S1 File shows that the pattern of the power comparisons by using significance level 5×10^−5^ is similar to that by using significance level 0.01 (Fig 1).

## Real data analysis

Chronic obstructive pulmonary disease (COPD) refers to a group of diseases that cause airflow blockage and breathing-related problems. The Genetic Epidemiology of COPD Study (COPDGene) is a multicenter observational study designed to identify genetic factors associated with COPD, to define and characterize disease-related phenotypes, and to assess the association of disease-related phenotypes with the identified susceptibility genes [28]. 10,192 participants (including 6,784 non-Hispanic Whites (NHW) and 3,408 African-Americans (AA)) are included in COPDGene. We selected 7 key quantitative COPD-related phenotypes and 4 covariates that are the same as those in Liang et al. [11]. The detailed description of these 7 phenotypes is in Table 3, and their correlation structure is given in Figure F in S1 File. The four covariates include Body Mass Index, Age, Pack-Years (one pack-year is defined as smoking one pack per day for one year), and gender. A set of 5,430 NHW across 630,860 SNPs were used in the analysis after excluding subjects with missing data in any of the 11 variables.

**Table 3:** Description of COPD-related phenotypes.

Phenotypes	Descriptions
Gas Trapping (GasTrap)	Air trapping at -856 Hounsfield units (HU) on expiratory chest CT scan
Exacerbation Frequency (ExacerFreq)	Number of COPD exacerbations during the year before study enrollment
Emphysema (Emph)	% Emphysema at -950 HU
Airway Wall Area (Pi10)	Square root of the wall area of a hypothetical 10 mm internal perimeter airway
Emphysema Distribution (EmphDist)	Log ratio of emphysema at -950 HU in the upper 1/3 of lung fields compared to the lower 1/3 of lung fields
Six Minute Walk Distance (6MWD)	Measure of exercise capacity
FEV1	Observed FEV1 (liters)/predicted FEV1 (liters), with predicted values from Hankinson reference equations

We apply WCmulP and other six methods to both original 7 phenotypes (Table 4) and the principal components (PCs) of the phenotypes (Table B in S1 File). PCs that explain less than 10^−6^ of the total variation are excluded. In this way, one PC is excluded and there are 6 PCs left. Using the first few PCs is also a dimension reduction method. Thus, using PCs of the phenotypes, WCmulP uses two dimension reduction methods: using the first few PCs and the weighted combination of those PCs. To identify SNPs significantly associated with the 7 COPD-related phenotypes and the top 6 PCs of the phenotypes, we use the genome-wide significance threshold of 5 × 10^−8^. There are total 16 SNPs that are significant under at least one method (Table 4 and Table B in S1 File). Those 16 SNPs have been reported being associated with the COPD-related phenotypes by previous studies [29–42]. From Table 4, we can see that MultiPhen identified the largest number of SNPs, 14 SNPs; WCmulP, SHet, Score, and CCA identified 13 SNPs; TATES identified 9 SNPs; and OB didn’t identify any SNPs, that’s likely because the true genetic effects of each SNP are heterogeneous for all phenotypes. From Table B in S1 File, we can see that using PCs of the phenotypes, WCmulP identified all of the 16 SNPs; MultiPhen identified 15 SNPs; SHet, Score, and CCA identified 13 SNPs; TATES identified 4 SNPs; and OB identified 3 SNPs. In summary, the number of SNPs identified by WCmulP is comparable to the largest number of SNPs identified by other tests; and using PCs of phenotypes, WCmulP is the only method that identified all 16 SNPs. The results of the real data analysis are consistent with our simulation results.

**Table 4:** Significant SNPs and the corresponding p-values in the analysis of COPDGene. The p-values of WCmulP are evaluated using 10^9^ permutations; the p-values of SHet are evaluated using 10^8^ permutations. The p-values of Score, MultiPhen, CCA, TATES, and OB are evaluated using asymptotic distributions. The grayed-out p-values indicate the p-values > 5 × 10^−8^.

Chr	Position	Variant identifier	WCmulP	SHet	Score	MultiPhen	CCA	TATES	OB
4	145431497	rs1512282	0	1.0E-08	1.90E-09	1.03E-09	1.69E-09	5.77E-09	0.339
4	145434744	rs1032297	0	0	5.55E-14	7.69E-14	6.52E-14	6.22E-13	0.452
4	145474473	rs1489759	0	0	1.11E-16	1.22E-16	1.11E-16	2.52E-16	0.483
4	145485738	rs1980057	0	0	1.11E-16	8.14E-17	0	9.35E-17	0.411
4	145485915	rs7655625	0	0	1.11E-16	9.13E-17	0	1.64E-16	0.478
15	78882925	rs16969968	0	0	1.91E-11	7.84E-12	1.32E-11	2.98E-08	0.986
15	78894339	rs1051730	1.00E-08	0	2.05E-11	8.16E-12	1.41E-11	2.63E-08	0.992
15	78898723	rs12914385	0	0	1.78E-12	1.48E-12	1.76E-12	5.14E-10	0.999
15	78911181	rs8040868	0	0	2.21E-12	2.59E-12	2.74E-12	2.40E-09	0.768
15	78878541	rs951266	2.00E-08	0	2.42E-11	1.02E-11	1.77E-11	5.17E-08	0.956
15	78806023	rs8034191	4.00E-08	1.0E-08	2.95E-10	7.74E-11	2.14E-10	1.02E-07	0.868
15	78851615	rs2036527	4.00E-08	1.0E-08	5.58E-10	1.77E-10	3.99E-10	1.56E-07	0.880
15	78826180	rs931794	4.80E-08	3.0E-08	3.13E-10	9.09E-11	2.35E-10	1.18E-07	0.913
15	78740964	rs2568494	7.18E-06	1.93E-06	1.22E-07	4.23E-08	1.05E-07	2.88E-05	0.269
15	78733731	rs17483721	8.12E-06	2.29E-06	2.26E-07	9.87E-08	2.11E-07	3.57E-05	0.308
15	78742376	rs17483929	8.15E-06	2.13E-06	1.65E-07	6.53E-08	1.50E-07	2.82E-05	0.347

## Discussion

In this article, we developed WCmulP to perform multivariate analysis of multiple phenotypes in association studies based on the following reasons: (1) complex diseases are usually measured by multiple correlated phenotypes in genetic association studies; and (2) there is increasing evidence showing that studying multiple correlated phenotypes jointly may increase powers for detecting genetic variants that are associated with complex diseases. Our results show that WCmulP has correct type I error rates and is either the most powerful test or comparable to the most powerful tests among the seven tests we considered. None of the other methods showed consistent good performances under the simulation scenarios. OB is the most powerful test when the genetic effects are homogeneous, while it loses power dramatically when genetic effects are heterogeneous; especially when opposite directions of the genetic effects exist. SHet, Score, MultiPhen, and CCA have similar powers and they are less powerful than WCmulP in most scenarios. TATES is more powerful only when the genetic variant affects a portion of phenotypes. In addition, in the real data analysis, WCmulP identified 13 (out of 16) significant SNPs, 1 SNP less than the largest number of identified SNPs; using PCs of phenotypes, WCmulP is the only method that identified all 16 SNPs. The real data analysis results show that WCmulP has excellent performance in identifying SNPs associated with complex disease with multiple correlated phenotypes such as COPD.

In the context of association studies, it is important to correct for population stratification (PS). PS refers to allele frequency differences between populations unrelated to the outcome of interest, but due to systematic ancestry differences. PS can cause seriously confounded associations if not adjusted properly [43, 44]. The principal component analysis (PCA) method [45–49] and linear mixed model (LMM) approach [50–52] have been used to adjust for population stratification. There are also other methods such as multidimensional scaling (MDS) [53], the robust PCA based on resampling by half means (RPCA-RHM) [54], and the robust PCA based on the projection pursuit (RPCA-PP) [54], which are extension methods of the PCA approach. PCA identifies several top principal components of the genotype data matrix and uses them as covariates in the association analysis. We propose to use PCA to control for PS in our proposed method when samples from different populations are involved. However, the performance needs further investigations.

One disadvantage of WCmulP is that the test statistic does not have an asymptotic distribution and a permutation procedure is needed to calculate its p-value, which is time consuming compared to the methods whose test statistics have asymptotic distributions. The running time of WCmulP with 1,000 permutations on a data set with 5,000 individuals and 20 phenotypes on a laptop with 4 Intel(R) Cores(TM) i7-4790 CPU @ 3.6GHz and 4 GB memory is no more than 0.15s. To perform GWAS, we can first select genetic variants that show evidence of association based on a small number of permutations (e.g. 1,000), and then a large number of permutations are used to test the selected significant genetic variants [21]. Furthermore, WCmulP cannot be used for rare variant association studies, although recent studies have shown that complex diseases are caused by both common and rare variants [50, 55–58]. How to extend WCmulP to rare variant association studies is our future work.

In our simulation studies, the numbers of phenotypes varied from 8 to 16 and the methods rely on all observations having fully observed phenotypes. However, in real data analysis, as the number of phenotypes increases the chance that missing at least one observation increases exponentially, especially in epidemiological and clinical research [59, 60]. There are several approaches to handle missing phenotypes: deletion-based methods, simple replacement methods, and imputation methods [59]. The most commonly used method for dealing with missing data is deletion-based method, in which observations with missing values are removed from the analysis [59]. However, removal of observations with missing values will reduce sample size, thus resulting in power losses [60]. The simple replacement methods replace the missing values with plausible values for the variable with missing values, such as the sample mean [8, 59]. It is a simple, unconditional method that does not depend on other variables. However, mean substitution approach may result in biased estimates where data are not missing completely at random [59]. Imputation is a more sophisticated approach that fills in missing values with predicted values using model-based methods or conditional imputation, including multiple imputation (MI), multivariate normal imputation (MVNI), and fully conditional specification (FCS) [59, 61–66]. In MI, the incomplete dataset is generated multiple times and missing values are replaced by values drawn from a posterior distribution according to a suitable imputation model that utilizes the rest of the data [59, 61]. MVNI fits a joint imputation model to all the variables containing missing values under the assumption that the variables follow a multivariate normal distribution [62, 63]. For each variable with missing values, FCS fits separate univariate regression models and iteratively cycles through the univariate regression models [64–66]. In our real data analysis, we removed 1354 observations with missing either phenotypes or covariates from 6784 samples. An alternative approach is to use mean substitution or imputation approaches to fill in the missing values.

## Supporting information

S1 File (PDF)
