# Supplementary material for: A novel method to test associations between a weighted combination of phenotypes and genetic variants
Source: PLoS One. 2018 Jan 12;13(1):e0190788. doi: 10.1371/journal.pone.0190788 (PMC5766098; doi:10.1371/journal.pone.0190788)

A Novel Method to Test Associations between a Weighted Combination of Phenotypes and Genetic Variants

Huanhuan Zhu, Shuanglin Zhang, Qiuying Sha*

Department of Mathematical Sciences, Michigan Technological University, Houghton, Michigan, USA

*Corresponding author

Supplementary Materials

Table A. The structures of γ and $\mathrm{cov}(y \mid x)$ for different numbers of factors when the number of phenotypes is 8.

	$R = 1$	$R = 2$	$R = 4$
γ	$\begin{pmatrix} 1 \\ 1 \\ 1 \\ 1 \\ 1 \\ 1 \\ 1 \\ 1 \end{pmatrix}$	$\begin{pmatrix} 1 & 0 \\ 1 & 0 \\ 1 & 0 \\ 1 & 0 \\ 0 & 1 \\ 0 & 1 \\ 0 & 1 \\ 0 & 1 \end{pmatrix}$	$\begin{pmatrix} 1 & 0 & 0 & 0 \\ 1 & 0 & 0 & 0 \\ 0 & 1 & 0 & 0 \\ 0 & 1 & 0 & 0 \\ 0 & 0 & 1 & 0 \\ 0 & 0 & 1 & 0 \\ 0 & 0 & 0 & 1 \\ 0 & 0 & 0 & 1 \end{pmatrix}$
$\mathrm{cov}(y \mid x)$	$\begin{pmatrix} 1 & c^2 & \cdots & c^2 \\ c^2 & 1 & \cdots & c^2 \\ \vdots & \vdots & \ddots & \vdots \\ c^2 & c^2 & \cdots & 1 \end{pmatrix}_{8\times 8}$	$\begin{pmatrix} 1 & c^2 & c^2 & c^2 & \rho c^2 & \rho c^2 & \rho c^2 & \rho c^2 \\ c^2 & 1 & c^2 & c^2 & \rho c^2 & \rho c^2 & \rho c^2 & \rho c^2 \\ c^2 & c^2 & 1 & c^2 & \rho c^2 & \rho c^2 & \rho c^2 & \rho c^2 \\ c^2 & c^2 & c^2 & 1 & \rho c^2 & \rho c^2 & \rho c^2 & \rho c^2 \\ \rho c^2 & \rho c^2 & \rho c^2 & \rho c^2 & 1 & c^2 & c^2 & c^2 \\ \rho c^2 & \rho c^2 & \rho c^2 & \rho c^2 & c^2 & 1 & c^2 & c^2 \\ \rho c^2 & \rho c^2 & \rho c^2 & \rho c^2 & c^2 & c^2 & 1 & c^2 \\ \rho c^2 & \rho c^2 & \rho c^2 & \rho c^2 & c^2 & c^2 & c^2 & 1 \end{pmatrix}$	$\begin{pmatrix} 1 & c^2 & \rho c^2 & \rho c^2 & \rho c^2 & \rho c^2 & \rho c^2 & \rho c^2 \\ c^2 & 1 & \rho c^2 & \rho c^2 & \rho c^2 & \rho c^2 & \rho c^2 & \rho c^2 \\ \rho c^2 & \rho c^2 & 1 & c^2 & \rho c^2 & \rho c^2 & \rho c^2 & \rho c^2 \\ \rho c^2 & \rho c^2 & c^2 & 1 & \rho c^2 & \rho c^2 & \rho c^2 & \rho c^2 \\ \rho c^2 & \rho c^2 & \rho c^2 & \rho c^2 & 1 & c^2 & \rho c^2 & \rho c^2 \\ \rho c^2 & \rho c^2 & \rho c^2 & \rho c^2 & c^2 & 1 & \rho c^2 & \rho c^2 \\ \rho c^2 & \rho c^2 & \rho c^2 & \rho c^2 & \rho c^2 & \rho c^2 & 1 & c^2 \\ \rho c^2 & \rho c^2 & \rho c^2 & \rho c^2 & \rho c^2 & \rho c^2 & c^2 & 1 \end{pmatrix}$

Table B. **Significant SNPs and the corresponding p-values in the analysis of COPDGene using the principal components (PCs) of phenotypes.** The p-values of WCmulP are evaluated using 10^9 permutations, the p-values of SHet are evaluated using 10^8 permutations. The grayed-out p-values indicate the p-values $> 5 \times 10^{-8}$.

Chr	Position	Variant identifier	WCmulP	SHet	Score	MultiPhen	CCA	TATES	OB
4	145431497	rs1512282	0	0	1.33E-09	9.28E-10	1.19E-09	2.85E-06	1.01E-04
4	145434744	rs1032297	0	0	1.18E-13	2.19E-13	1.40E-13	2.58E-09	6.91E-07
4	145474473	rs1489759	0	0	2.22E-16	3.14E-16	2.22E-16	3.19E-12	2.32E-08
4	145485738	rs1980057	0	0	1.11E-16	2.16E-16	1.11E-16	9.21E-13	1.72E-08
4	145485915	rs7655625	0	0	2.22E-16	2.68E-16	1.11E-16	1.80E-12	1.63E-08
15	78882925	rs16969968	0	0	7.84E-12	3.93E-12	5.42E-12	6.56E-07	1.99E-03
15	78894339	rs1051730	0	0	8.23E-12	4.02E-12	5.63E-12	5.43E-07	1.35E-03
15	78898723	rs12914385	0	0	5.60E-13	5.10E-13	5.53E-13	4.94E-07	4.64E-05
15	78911181	rs8040868	0	0	8.47E-13	1.10E-12	1.05E-12	3.42E-07	2.58E-04
15	78878541	rs951266	0	0	9.82E-12	4.85E-12	7.16E-12	6.65E-07	2.96E-03
15	78806023	rs8034191	0	0	1.04E-10	3.19E-11	7.56E-11	4.19E-06	6.25E-03
15	78851615	rs2036527	0	0	2.13E-10	7.79E-11	1.52E-10	9.02E-06	5.36E-03
15	78826180	rs931794	0	0	1.04E-10	3.29E-11	7.71E-11	2.47E-05	1.66E-02
15	78740964	rs2568494	4.10E-08	6.0E-08	6.97E-08	2.58E-08	6.01E-08	3.01E-04	3.69E-02
15	78733731	rs17483721	5.00E-08	2.2E-07	1.25E-07	6.10E-08	1.17E-07	2.86E-04	2.64E-02
15	78742376	rs17483929	4.00E-08	9.0E-08	8.89E-08	3.89E-08	8.08E-08	2.94E-04	3.28E-02

Figure A. Power comparisons of the seven methods as a function of β for the six models using the principal components (PCs) of phenotypes. The total number of phenotypes is $K = 8$, $c^2 = 0.5$, $\rho c^2 = 0.1$, and $MAF = 0.3$. The p-values of WCmulP and SHet are evaluated using 1,000 permutations. The power of all of the seven methods is evaluated using 1,000 replicated samples at a significance level of 0.01.

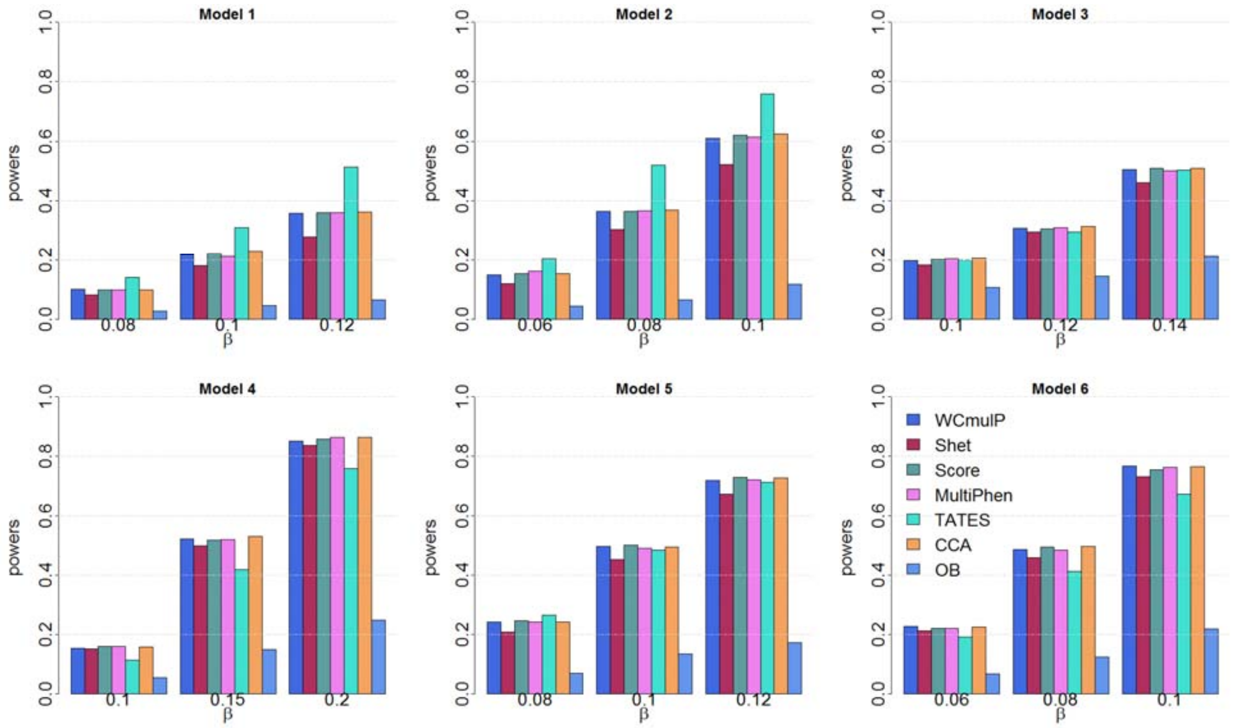

Figure B. Power comparisons of the seven methods as a function of β for the six models using the principal components (PCs) of phenotypes. The total number of phenotypes is $K = 16$, $c^2 = 0.5$, $\rho c^2 = 0.1$, and $MAF = 0.3$. The p-values of WCmulP and SHet are evaluated using 1,000 permutations. The power of the seven methods is evaluated using 1,000 replicated samples at a significance level of 0.01.

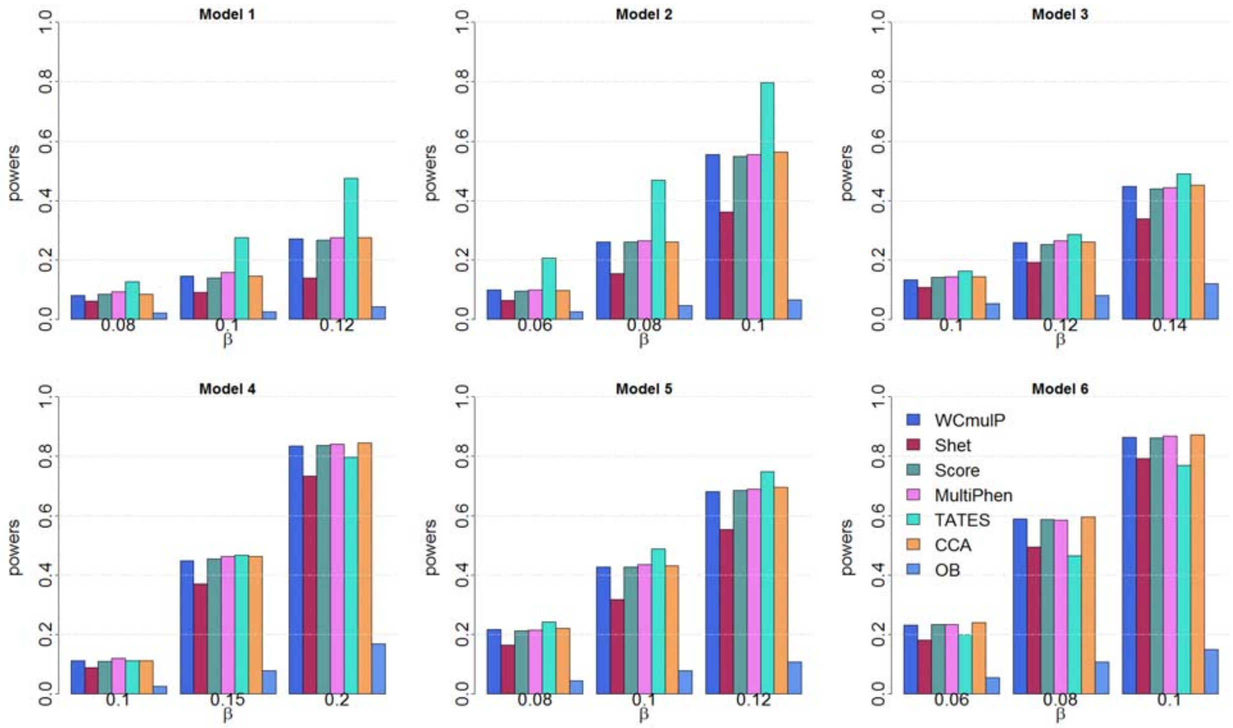

Figure C. **Power comparisons of the seven methods as a function of c^2 for the six models using the principal components (PCs) of the phenotypes.** The total number of phenotypes is $K = 8$, $\rho c^2 = 0.1$, $\beta = 0.1$, and $MAF = 0.3$. The p-values of WCmulP and SHet are evaluated using 1,000 permutations, the p-values of other methods are evaluated using asymptotic distribution. The power of all of the seven methods is evaluated using 1,000 replicated samples at a significance level of 0.01.

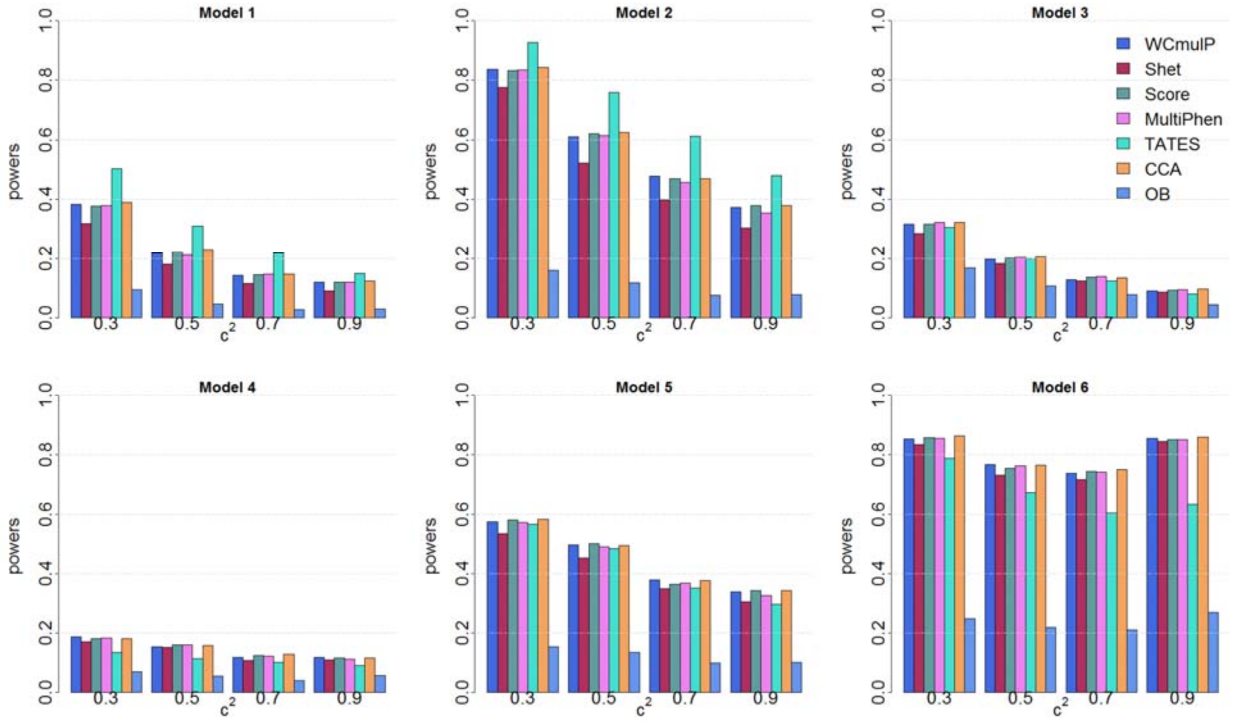

Figure D. **Power comparisons of the seven methods as a function of c^2 for the six models using the principal components (PCs) of the phenotypes.** The total number of phenotypes is $K = 16$, $\rho c^2 = 0.1$, $\beta = 0.1$, and $MAF = 0.3$. The p-values of WCmulP and SHet are evaluated using 1,000 permutations, the p-values of other methods are evaluated using asymptotic distribution. The power of all of the seven methods is evaluated using 1,000 replicated samples at a significance level of 0.01.

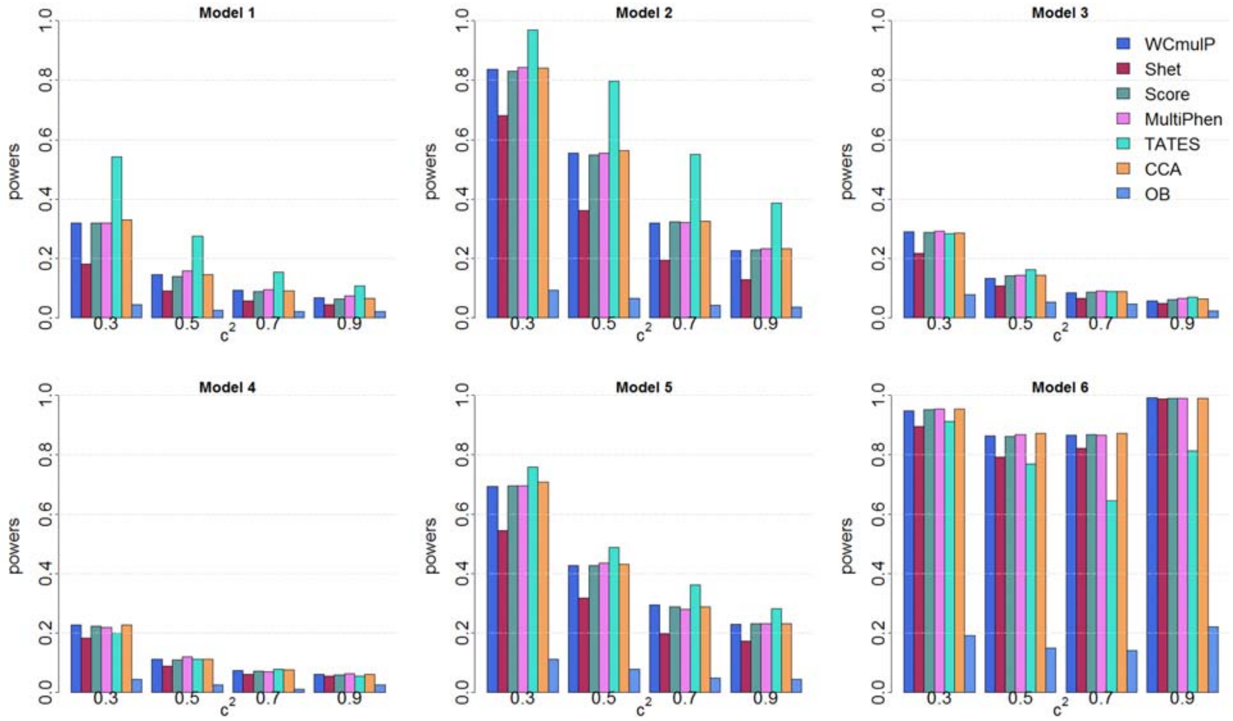

Figure E. **Power comparisons of the seven methods as a function of β for the six models.** The total number of phenotypes is $K = 8$, $c^2 = 0.5$, $\rho c^2 = 0.1$, and $MAF = 0.3$. The p-values of WCmulP and SHet are evaluated using 100,000 permutations. The power of all of the seven methods is evaluated using 1,000 replicated samples at a significance level of 5×10^{-5}.

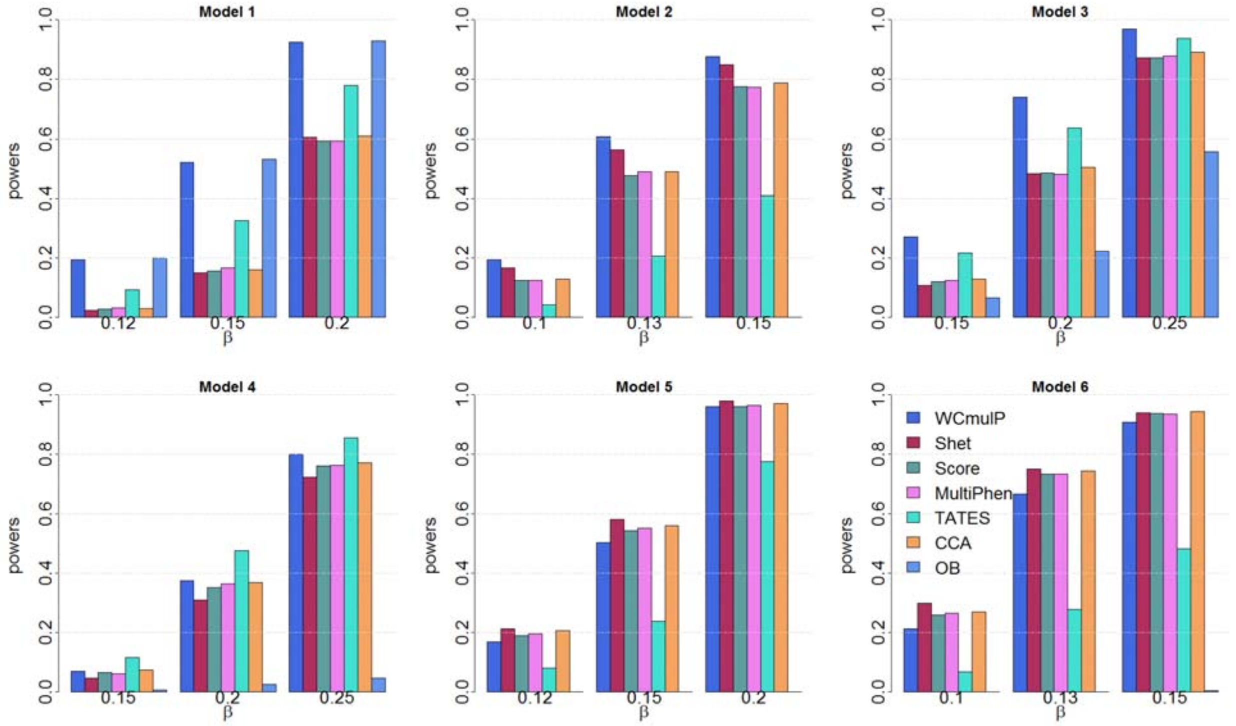

Figure F. The correlation matrix plot of the 7 COPD-related phenotypes.

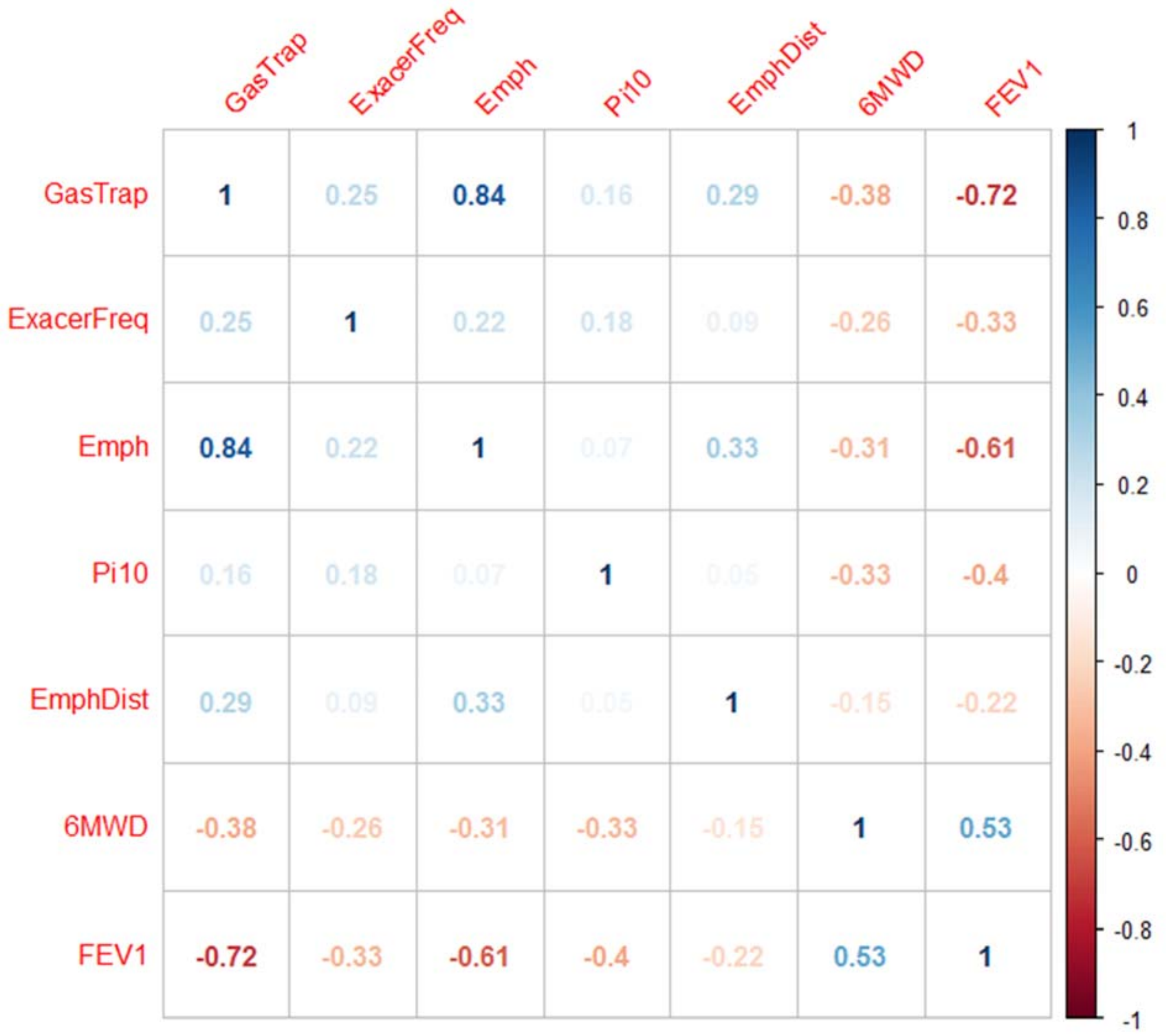

Supplement: S1 File — (PDF) [file pone.0190788.s001.pdf]
